# The effect of ionic strength on oil adhesion in sandstone – the search for the low salinity mechanism

**DOI:** 10.1038/srep09933

**Published:** 2015-04-22

**Authors:** E. Hilner, M. P. Andersson, T. Hassenkam, J. Matthiesen, P. A. Salino, S. L. S. Stipp

**Affiliations:** 1 Nano-Science Center, Department of Chemistry, University of Copenhagen, Denmark; 2 BP Exploration, Sunbury, U.K

## Abstract

Core flood and field tests have demonstrated that decreasing injection water salinity increases oil recovery from sandstone reservoirs. However, the microscopic mechanism behind the effect is still under debate. One hypothesis is that as salinity decreases, expansion of the electrical double layer decreases attraction between organic molecules and pore surfaces. We have developed a method that uses atomic force microscopy (AFM) in chemical force mapping (CFM) mode to explore the relationship between wettability and salinity. We functionalised AFM tips with alkanes and used them to represent tiny nonpolar oil droplets. In repeated measurements, we brought our “oil” close to the surface of sand grains taken from core plugs and we measured the adhesion between the tip and sample. Adhesion was constant in high salinity solutions but below a threshold of 5,000 to 8,000 ppm, adhesion decreased as salinity decreased, rendering the surface less oil wet. The effect was consistent, reproducible and reversible. The threshold for the onset of low salinity response fits remarkably well with observations from core plug experiments and field tests. The results demonstrate that the electric double layer force always contributes at least in part to the low salinity effect, decreasing oil wettability when salinity is low.

When oil is produced from a reservoir, the first 5 to 10% comes as a result of pressure reduction. To produce more, oil reservoirs are flooded with whatever water is at hand, such as sea water (salinity 36,500 ppm total dissolved solids, TDS) or water from a nearby rock formation (where salinity can be 200,000 ppm or more). However, even after water flooding, significant quantities of oil remain. Flooding with water of lower salinity (< 6,000 ppm) increases oil recovery from sandstone reservoirs, as demonstrated by a number of core and field tests^1^,^2^,^3^,^4^,^5^,^6^,^7^. This phenomenon is called “low salinity enhanced oil recovery” (EOR). The mechanism responsible for the oil release is still debated and is likely a combination of many processes. One hypothesis is that low salinity water alters the wettability of the pore surfaces in contact with the oil. Polar functional groups on the molecules in the oil adsorb on surfaces, rendering them oil wet. With low salinity water flooding, some of these molecules desorb, the surfaces become more water wet and oil is released. Several adsorption/desorption routes have been proposed^5^,^8^,^9^.

Sandstone consists mainly of quartz, feldspar and clays. The minerals have a point of zero charge that is lower than the typical pH in a sandstone reservoir. The surfaces are thus negatively charged and the surface charge changes as a function of solution pH. Austad et al.^5^ suggested a mechanism where ions, in particular Ca^2+^, as well as the polar oil components, adsorb to the negatively charged surface sites. They proposed that when low salinity water passes through the pores, adsorbed cations are substituted by H^+^, leading to a local pH increase near the surface and desorption of polar oil components through acid-base reactions. This would result in a wettability evolution toward more water wet. Lager et al.^8^ suggested that multicomponent ion exchange (MIE) takes place at the pore surface with low salinity flooding so polar compounds desorp from the surface when the low concentration of ions promotes breaking of mineral-cation-oil component bridges. This would also change the wettability toward more water wet conditions. Nonpolar oil compounds are thought to be adsorbed only by van der Waals (VDW) forces^8^,^9^ through Debye interaction and London dispersion. The VDW force and the force originating from the electrical double layer (EDL), which is formed at the interface of water and a surface, contribute to the interaction between any molecule and a charged surface. Desorption of organic molecules is favoured by the expansion of the electric double layer, which occurs when the ionic strength decreases^9^,^10^. This balance between VDW and EDL forces can be described qualitatively, using Derjaguin, Landau, Verwey and Overbeek (DLVO) theory^11^. The tip-surface interaction can alternatively be described by considering the interfacial energies between tip, surface and medium^12^. Lee et al.^13^ investigated systems of pure silica (amorphous Ludox® AS particles) and model hydrocarbons in water using neutron and X-ray scattering and elipsometry. Their data were fit well with a model that incorporated a thin water layer between the silica and the hydrocarbon, where water layer thickness varied in proportion to ionic strength and ion type.

Detailed understanding of the mechanisms behind oil release, in response to change in salinity, would provide a tool for estimating if a reservoir would respond well to low salinity EOR as well as which solution conditions would work best. Too low ionic strength causes some types of clay minerals to swell, which can lead to pore clogging, risking serious reservoir damage. A balance where as much oil as possible can be produced without reservoir damage is the goal. Macroscopic core plug tests give valuable hints but nanometer scale information is essential for testing the conceptual models that are constructed using macroscopic data.

To measure the adhesion force between surfaces and molecules with specific functional groups that are expected to be found in oil, we can use atomic force microscopy (AFM) where we functionalise the AFM tip with the molecule of interest. Although the composition of crude oil is very complex, and each crude has a different composition, there is a set of functional groups that are common. The gold coated AFM tip can be functionalized by forming a self assembled monolayer of molecules with thiol on one end and the desired group on the other. This approach is well known and tested in materials science^14^,^15^. The thiol forms a strong bond with the gold surface^16^,^17^,^18^ and because of intermolecular interaction, the molecules form a brush on the surface^19^. For the experiments described in this paper, we used 1-undecanthiol, which gives a nonpolar, -CH_3_ termination to the AFM tip so it serves as a tiny, model, oil droplet. We chose to work with methyl terminated tips because then the adhesion force we measure is directly linked to the wettability of the surface^20^. Change of wettability of the pore surfaces is, as stated by Lager et al.^8^ and Austad et al.^5^, is central to the low salinity mechanism. Both -CH_3_ and -COO(H) terminated tips have been used previously, to investigate the effect of changing salinity, on surface adhesion in sandstone and chalk^20^,^21^,^22^,^23^. AFM has also been successfully used to study interaction forces between bitumen and grains in oil sands^24^,^25^.

The AFM experiments were performed in solution, measuring adhesion as a function of salinity. We use a method called chemical force mapping (CFM), which provides *change* in adhesion as a function of location on the surface. Force mapping has previously been described on natural surfaces and has been used by our group on chalk^26^, sandstone^20^,^23^ and on model surfaces^27^,^28^ that were designed to explore the behaviour of the minerals that are present in sandstone^21^. We started with a solution of artificial formation water (AFW) and then sequentially mixed it with diluted artificial sea water (ASW) to provide solutions that ranged in salinity from 28,500 ppm total dissolved solids (TDS) to 1,500 ppm. We have previously demonstrated a low salinity response on the surfaces of quartz grains from sandstone and specifically that organic material on the mineral surface plays an important role in the low salinity effect^23^. In this paper, we take the concepts a step further, with the aim of quantifying the relationship between solution salinity and the low salinity response. We decreased ionic strength sequentially and then increased it again by mixing aliquots of AFW so we could i) identify the threshold where the low salinity effect begins to appear and ii) elucidate how large a role, the electric double layer expansion plays in the observed adhesion behaviour.

## Experimental details

### Samples and solutions

For the experiments described in this paper, we used a sample of sandstone (Core C) from an oil reservoir that had a reasonable low salinity response, i.e. ~ 9% increased oil production, determined from traditional core plug tests. This falls within the usual range expected for low salinity flooding in sandstone. The core had been preserved in kerosene. We chipped small pieces from it, using the tip of a scalpel blade. The kerosene evaporated when the rock chips were heated on a hot plate at 70–90°C for ∼ 30 min. Single grains were taken from the chips and glued to a glass microscope cover slip using a thin layer of two component epoxy (Danalim 335). It is important for the grains to stick but not to sink into the glue. The epoxy was allowed to cure for at least 24 hours, thus minimising the risk of reaction with the saline solutions. The cover slips with attached grains were inspected using an optical microscope, to identify grains with surfaces that were sufficiently flat to be suitable for AFM imaging. We chose quartz grains that showed clear evidence of recrystallisation during diagenesis, which we used as indication that they had been present at pore surfaces and that they were in fact quartz and not feldspar. We know that this type of quartz grain surfaces on average exhibit a low salinity response, from previous work^20^,^21^,^23^, Recent studies have made it quite clear that these quartz grain surfaces are not clean, pure mineral surfaces but rather, that they have a variable amount of organic material on them^23^.

We made two stock solutions for our experiments, artificial formation water, AFW, which matched the composition of the water present in the rock formation where our sample was collected and the other, low salinity artficial sea water, ASW, that was dilute enough to represent a solution that could be used for low salinity flooding. The composition of AFW was ~ 27,000 ppm NaCl, ~ 170 ppm KCl, ∼ 190 ppm MgCl_2_ and ~ 730 ppm CaCl_2_. Total salinity expressed as mg/kg TDS was ~ 28,000 ppm. The diluted ASW was made from a recipe for artificial seawater^29^ that we adapted, to leave out SO_4_ and HCO_3_. TDS was ~ 1,500 ppm and its composition was ~ 1,200 ppm NaCl, ~ 40 ppm KCl, ~ 210 ppm MgCl_2_ and ~ 50 ppm CaCl_2_. We used no buffers, to avoid contamination of surfaces and tip with extraneous compounds. pH was not adjusted but was measured after preparation of the AFW, ASW and diluted ASW solutions and was between 5.3 and 5.5 for all solutions. This pH is typical for many sandstone reservoirs.

### AFM tips

For standard imaging with AFM tapping mode, we used Olympus AC 160 tips with a nominal cantilever spring constant of 40 nN/nm. To prepare the functionalised tips, we used gold coated Biolevers that had a nominal spring constant of 30 pN/nm. The tips were cleaned with UV/ozone for 20 min before immersing them in an ethanol solution containing 2 mM 1-undecanethiol, HS(CH_2_)_10_CH_3_.

We know that these functionalised tips are robust enough to withstand service through thousands of force curves. First, the use of self assembled monolayers (SAMs) produced with molecules that are attached to gold surfaces using thiols is well established^19^. Second, the experiments presented in this study elaborate on previous results^23^ where we switched directly between high and low salinity conditions, using an identical setup and samples from the same core. In that study, we saw a consistent and reproducible decrease in adhesion in response to changed solution composition that is completely consistent with the results of this study. In the previous work, we also demonstrated that there was no difference in adhesion that correlated with which solution we began with. Therefore, it is not possible to explain our results with wear on the tip.

After a minimum of 24 hours in the thiol solution, the tips were removed and rinsed with clean ethanol. The actual spring constant, determined from a fit to the thermal spectrum, was between 18 and 20 pN/nm. We used a new tip for each experiment but we did not change the tip during an experiment so the tip-sample relationship would remain constant. We expect that the -CH_3_ terminated tip serves as a good model for a crude oil with a low to medium total acid number (TAN) < 1^30^.

### Experimental procedure

The glass cover slip, with the attached quartz grains, was mounted in an MFP-3D (Asylum Research) AFM, an instrument optimised for force mapping. To start the experiments, we immersed the sample in about 3 ml of artificial formation water to equilibrate the mineral surfaces with the solution so the surface composition would resemble that initially present in the reservoir. A force map using the -CH_3_ functionalised tip on a quartz particle was then collected, using a routine written by S. Vinzelberg (Atomic Force). We then diluted the AFW in a series of steps. Each time, we removed a small amount of the solution from the fluid cell, typically 0.5–2 ml, to avoid the tip drying out. Then we replaced it with the same amount of diluted ASW, to sequentially decrease the ionic strength. All fluids were added or removed with a syringe, which was weighed before and after, to record the mass of solution exchanged. After each such dilution, adhesion data were collected. An interesting benefit with this approach is the opportunity to gain new information about the effects of mixing low salinity water into the water initially present in the reservoir, as would happen in field applications^31^. After force maps were collected for about six or seven dilutions, salinity was increased again by sequential additions of the AFW. This allowed us to check whether the effect was reversible.

From the mass of solution added or removed, the solution composition could be determined. Each sample removed from the fluid cell was analysed using atomic absorption spectroscopy (AAS). For the calculations, we used a density of 1.03 g/ml for the AFW and 1 g/ml for the low salinity ASW. An experiment typically lasted for ∼ 10 hours and during that time, a small amount of water evaporates from the fluid cell so salinity increases slightly. The salt remains as the water vaporises so we compensated by adding enough ultrapure deionised (DI) water (MilliQ, resistivity 18 MΩ/cm) to keep the fluid at precisely the same level in the fluid cell during the experiments. The total amount of ultrapure water added over the period of the experiment was 1.1 to 1.3 ml.

Force curves were recorded over areas of 5 µm × 5 µm, with grids of 50 × 50 pixels. Each force vs. distance curve collects information from the complete cycle of tip approach and retraction, as described previously by Hassenkam et al.^26^ and as shown in Figure S1 in the Supporting Information. The adhesion force was calculated from the minimum of the retraction curve relative to the flat part of the approach curve. Using the flat part of the approach curve as baseline gives more consistent adhesion force calculations on rough surfaces, even though the drag force introduced by cantilever displacement through the solution is included in the calculated adhesion force^26^. This is justifiable because the drag force is essentially constant at 10 to 20 pN, which would add a negligible amount to the adhesion measurement and more important, we are interested in the *change in* adhesion rather than the *absolute* value and this small amount, being constant, would cancel out.

### Theoretical details

There is an attractive component to the adhesion force between the AFM tip and the rock surface that comes from van der Waals forces and hydrophobic interactions. This force depends on the local properties of the surface but it does not depend on salinity^32^. It is most likely heterogeneously distributed organic material that gives rise to the adhesion between the surface and the hydrophobic tip^23^ because the adhesion between a -CH_3_ tip and a clean quartz surface is nearly 0^28^. This extremely low adhesion for a clean quartz surface means that the attractive forces between nonpolar oil and quartz are not strong enough to overcome the attractive forces between water and quartz, consistent with contact angle measurements on clean quartz^33^. Because of the heterogeneous distribution of organic material on the surface, this attractive force can be expected to vary between force maps taken on different areas of the sample. There is also a certain amount of variability in the size and shape of the tip that is essentially uncharacterisable. This also contributes to differences in the absolute adhesion measured on a surface. This is not a problem however, because it is the measure of the difference in adhesion at the same location, with the same tip, after the solution concentration is changed that is the goal of these experiments. Thus, although absolute adhesion can vary from one experiment to the next, and cannot be compared directly, the *change* in adhesion as a function of solution salinity is a valid parameter, that can be compared.

When ions are present in the solution and the tip or surface is charged, an additional term has to be added to the interaction force that comes from the electric double layer (EDL)^32^,^34^. We use an expression from Butt^34^,^35^ for the force exerted on the tip by the EDL under constant charge conditions:


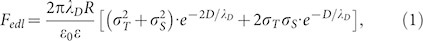
where R represents the tip radius, ε_0_, the dielectric permittivity of vacuum, ε, the relative permittivity of the medium, D, the tip/sample distance, σ_T_ and σ_S_, the surface charge densities of the tip and surface and λ_D_, the Debye length, which is:


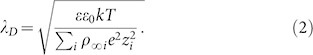
 In this case, ε represents the relative permittivity for salt water, ε_0_, the vacuum permittivity, k, Boltzmann's constant, T, temperature, e, the elemental charge, 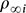, the number density (m^−3^) of the ion, i and z represents the valence of ion, i. Equation (1) is an approximation for a spherical tip and a flat substrate. Our tips are pyramidal shaped so a more stringent expression^25^ could be used, but we can take advantage of a simpler approximation. The Debye length and the distance between the tip and the substrate are both on the order of 1 nm at contact. The radius of the tip is roughly 30 nm, which is much larger than 1 nm so the contribution from the cone shaped part of the tip is negligible and the assumption of a sphere at contact becomes a very good approximation.

Our -CH_3_ terminated tip carries no surface charge, i.e. σ_T_ = 0, so the EDL force between the tip and sample simplifies to:


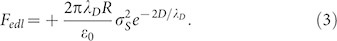


This relationship is strictly valid only where D ≥ λ_D_ and for ionic strength less than ~ 100 mM. However, in a recent paper, the charge state of silica was determined at ionic strength comparable to ours from adhesion force data, i.e. similar to our method. These results were consistent with previous results obtained at lower ionic strength by fitting force vs. separation curves. This provides confidence that EDL theory gives reasonable results even for the short Debye lengths at ionic strengths stronger than 100 mM^36^. For distances significantly smaller than the Debye length, the continuum theory is expected to fail because the size and discrete nature of the ions is important. The total force between the tip and the surface is the sum:


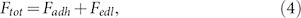
where F_adh_ represents the salinity independent attractive van der Waals force and hydrophobic interaction; the expression for F_edl_, is described by Eq. 3.

## Results and discussion

### Solution composition

**Figure 1:**
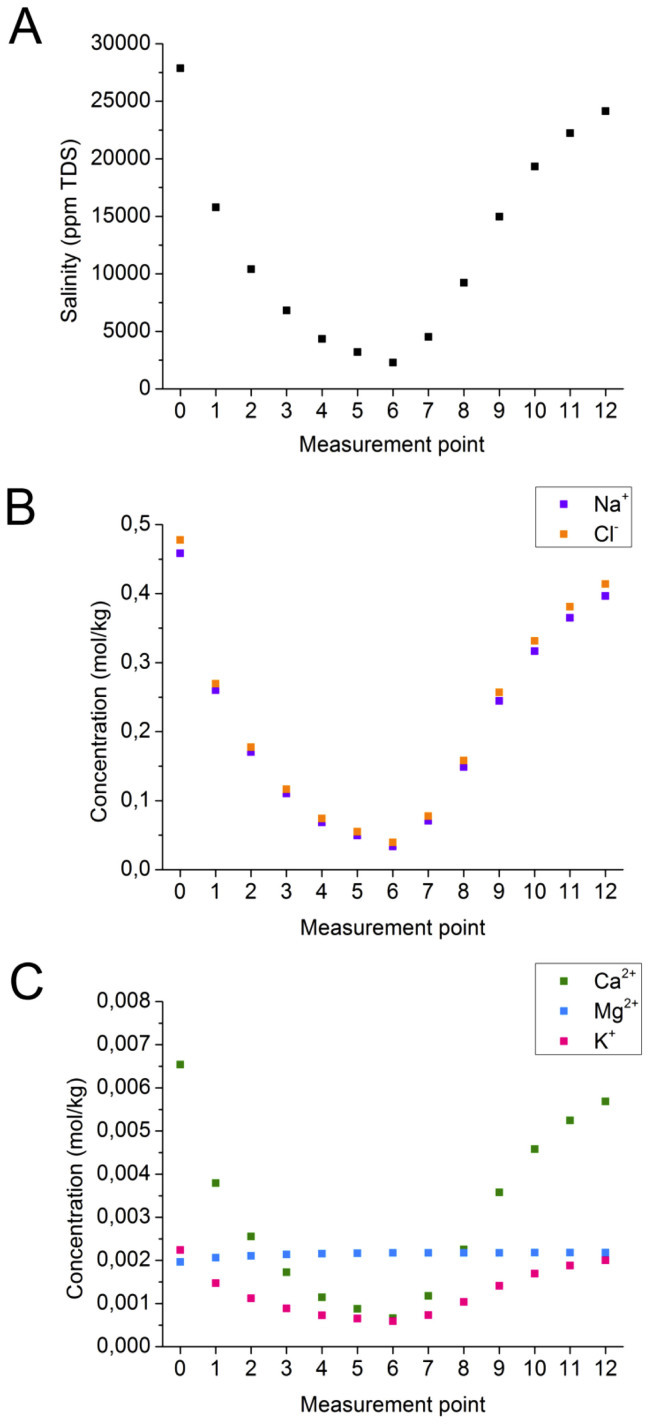
Experiment 1, data for A) salinity, B) and C) ion concentrations. Point 0 in all experiments corresponds to pure, artificial formation water (AFW). Points 1 through 6 were made by adding aliquots of low salinity artificial sea water (ASW). Points 7 to 12 represent solutions where aliquots of AFW were added to increase salinity.

We collected force measurements through the full sequence of salinity change, starting with saline water, dilution and return to full salinity, during three, separate experiments. In each, data were recorded for 12 or 13 different solution concentrations. Salinity was derived from the initial quantities of saline solution added together with the mass of solutions removed and added. The composition plots for Experiment 1 are presented in Figure 1. Measurement point 0 was made in pure AFW. Points 1 through 6 were made in AFW diluted with low salinity artificial sea water (ASW). For Points 7 through 12, salinity was increased again by introducing aliquots of AFW. The composition plots for Experiments 2 and 3, which are Figures S2–S4 in Supporting information, are very similar.

The ratio of the various ions in the diluted ASW, intended to mimic the diluted sea water that is often used for low salinity reservoir flooding, is not the same as the formation water so there is a difference in how the concentrations of the various ions change throughout the experiments. Na^+^ and Cl^−^ are quite concentrated (Fig. 1B) compared with Ca^2+^, Mg^2+^ and K^+^ (Fig. 1C). Mg^2+^ remains essentially constant because its concentration in low salinity ASW is almost the same as in the formation water from the reservoir where this sample was taken. As a consequence, our results could differ from previous experiments that have used diluted ASW. The different ratio of divalent to monovalent ions would not influence the EDL expansion but ion exchange at the solid surface could certainly be affected by different solution compositions.

### Sample surface morphology

**Figure 2:**
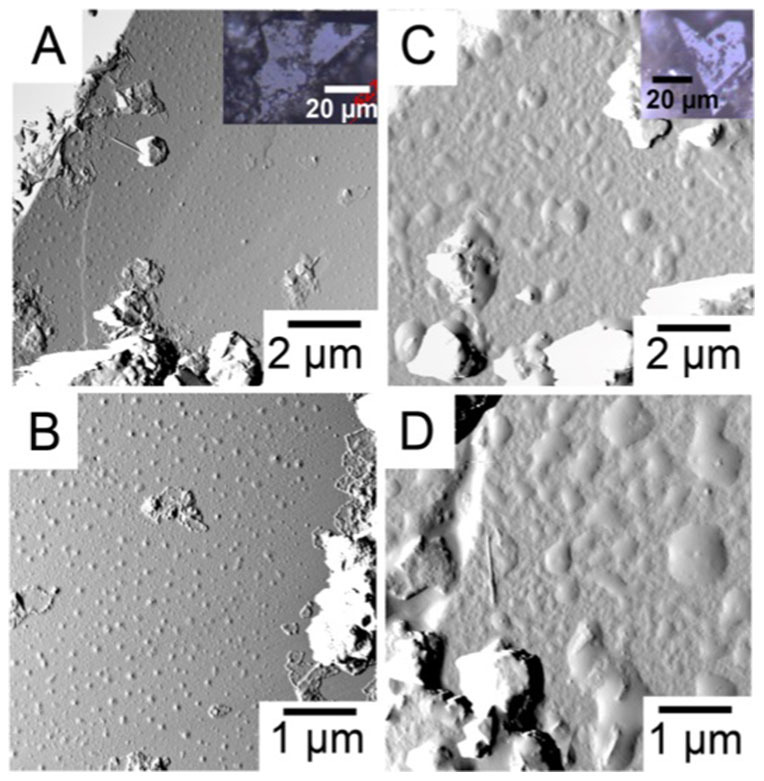
Tapping mode AFM images, showing topography, from A) and B) Sample 1; C) and D) Sample 2. The contrast results from changes in oscillation amplitude, as the tip gets closer to the surface. This imaging mode enhances feature edges. These surfaces have not been imaged precisely over the same areas as where the force maps were recorded but they are representative for the types of surface morphology that we generally observe on quartz grain surfaces from sandstone. The insets are images from an optical microscope for A) Sample 1 and C) Sample 2.

Experiments 1 and 2 were conducted on two different areas of the same quartz grain (Sample 1). Experiment 3 was conducted on a different quartz grain (Sample 2). We identified the sand grains as quartz by their hexagonal crystal form, i.e. the angles between crystal faces, observed by optical microscopy and we confirmed their identity by scanning electron microscopy (SEM) with energy dispersive X-ray spectroscopy (EDXS) after the AFM experiments were completed. Figure 2 shows typical surface character from the two samples. In some areas, there are clusters of material with the crystal form of clay nanoparticles. We expect the adhesion behaviour of clay to be different than that of quartz unless, for example, organic material covers the whole surface^23^. Also common are features that resemble droplets, such as could be formed by remnant organic material. On Sample 1, we see small bumps that are distributed over the surface. On Sample 2, the bumps are larger and somewhat coalesced, implying a higher concentration of this material. The nature of the surface changes over the scale of a few nanometers. These features could be organic material, which certainly affects the surface energy at the mineral-water interface and they modify the mineral-tip interaction. Consequently, the surface features influence the part of the adhesion force that does not depend on the ionic strength (Eq. 1). We expect the ionic strength component to be similar for all surfaces but the effect of the surface features, such as those observed in Figure 2, would certainly be different for the three experiments, which were conducted on different areas on quartz crystals. This would give rise to different absolute adhesion for each experiment.

### Force measurements

**Figure 3:**
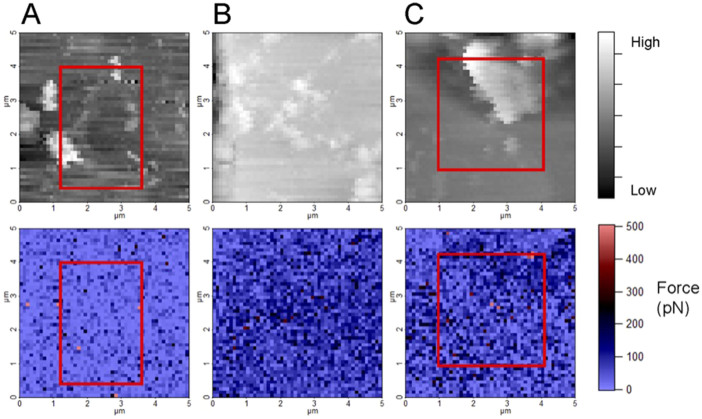
Topography images (upper row) and the corresponding force maps (lower row) for A) Experiment 1 (Sample 1), B) Experiment 2 (Sample 1), **C**) Experiment 3 (Sample 2). The red squares in A) and C) outline the areas that were visible in all maps and that were used for determining average adhesion.

Figure 3 shows typical maps made from the force curve data derived from the 2,500 force distance curves that were collected over the selected areas. More information about force curves and force mapping is described by Hassenkam and colleagues^26^. For the top row of images in Fig. 3, the tip's position at the point of maximum force was taken from each of the curves (such as Fig. S1) to construct maps that resemble the topography images produced in tapping mode, such as in Figure 2, but with much poorer resolution. The poorer resolution is a result of only having 2,500 (50 × 50) pixels per map compared with more than a hundred times more points (512 × 512) for the tapping mode images, as well as the measurement mode that was used to acquire the data points. The adhesion force maps in the bottom row are made from the force offset from the same set of data so they show precisely the same area, collected at the same time. The features visible on the topography maps are used to calibrate for position, to ensure that the force maps are acquired at the same location over the entire 10 hours of the experiment. In some cases, such as for Experiments 1 and 3, the imaging area migrates slightly over the 10 hours. Drift is normal in AFM mapping but to compensate for drift, we selected a region from inside the 5 × 5 μm^2^ area that was present in the field of view for the full set of 13 images. The regions selected for deriving average adhesion for Experiments 1 and 3 are shown in Fig. 3A and C. There was minimal drift in Experiment 2 so in this case the whole area could be used.

**Figure 4:**
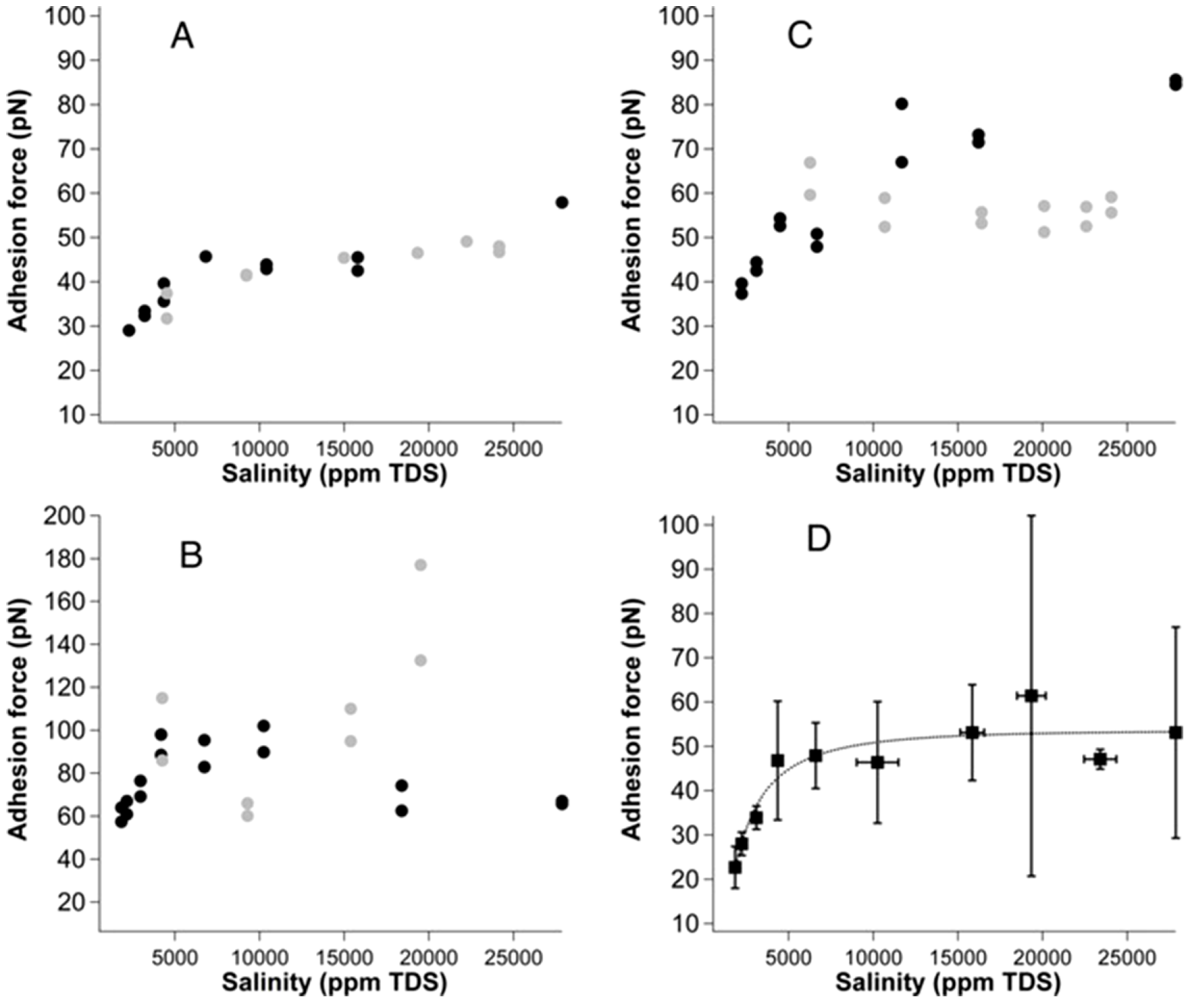
Adhesion force plotted as a function of salinity: A) Experiment 1 (Sample 1), B) Experiment 2 (Sample 1) and C) Experiment 3 (Sample 2). Black points represent a series of experiments for decreasing salinity; grey points represent a series for increasing salinity: D) a fit, using Eq. 4, for all of the data from all three experiments. The data were offset with a constant so that adhesion at ~ 3000 ppm was set equal for the three experiments.

The average adhesion, plotted as a function of salinity for all experiments, is shown in Figure 4. The data acquired while salinity decreased have been coloured black whereas those acquired as salinity increased are shown in grey. Adhesion was directly related to salinity, it was reversible and hysteresis was minimal. The standard deviation for each adhesion measurement from the force curves is 4 to 5 pN, i.e. well within the size of the data points, but the variability of adhesion force over the surface is considerable, because the surface is heterogeneous. Adhesion depends on the material that the tip interacts with on the sand grain, which could be quartz, clay or organic material. We have not added bars to represent the deviation of the data because this would imply that we assume the surfaces to be homogeneous – but they are not. The adhesion variation in the force maps reflects variability in adhesion, not uncertainty.

In all experiments, there is a clear decrease in adhesion when the salinity falls below a threshold of ~ 5,000 ppm. The trend is particularly clear for the figure made with the pooled data set, plotted in Figure 4D. The larger spread between increasing and decreasing salinity in Figure 4B and C probably results from a change in the character of the surface or the tip during the experiment. This is certainly not unusual in experiments on natural samples where abundant organic material is present.

The absolute adhesion for the measured regions in Experiments 1 and 3 (Figure 4A and C) is roughly at the same level, whereas adhesion from Experiment 2 (Figure 4B) is about a factor of two higher. A difference in the surface heterogeneity, in particular an uneven distribution of clay nanoparticles and organic material, can explain a difference in absolute adhesion for experiments on different regions on the sample. The adhesion work, i.e. surface energy, depends strongly on the amount and type of organic material adsorbed on the mineral surface^32^. In another study, this lead to large variability in the absolute adhesion on surfaces of quartz grains from this same core plug^23^. In summary, although there are differences between the absolute adhesion for the three experiments, the trend is clear. As salinity decreases from the AFW toward low salinity ASW, adhesion remains relatively constant until the threshold is reached. From there, we see a pronounced decrease, dropping by about 50% as salinity decreases from 5000 ppm to 2000 ppm.

### Comparison with theory

If we shift the adhesion force results for each experiment by a constant that accounts for the tip and surface inhomogeneities, so the force at low salinity is constant for all three experiments, the salinity independent differences are removed. The data superpose well (Fig. 4). All experimental results for each salinity cluster into single points. We used the following procedure: We sorted the data from low to high salinity. We compared the salinity point by point and wherever the relative difference was more than 10%, a new pooled data point was created. The range of the salinities making up each point is shown as the horizontal uncertainty bars in Figure 4D. The standard deviation of the adhesion force for each pooled data point is shown as vertical uncertainty bars in Figure 4D. An outlier test^37^, applied for each pooled data point, failed to identify any outliers. Note that the error bars do not represent measurement uncertainty as such, rather the variations in adhesion are caused by changes in the system, such as transfer of material to the tip or removal of material from the surface by the tip or addition of material to the surface when the solution is exchanged. Using the pooling procedure with an outlier test allowed us to include all data and not worry about the slightly different trend when the salinity is increased and decreased (Fig. 4B and C). Even though Figure 4B does not exhibit as clear an adhesion drop at low salinity as the other experiments, inclusion of these experimental data into the pool of Figure 4D strengthens the correlation and the data set responds negatively to the outlier test, thus strengthening the conclusions. We used Eq. 4 to fit the pooled data in Figure 4D and calculated Debye length from the solution composition and salinity according to Eq. 2. Tip radius, R, is given by the manufacturer to be 30 nm. The relative permittivity of the solution is assumed to be that of water, i.e. 80. We also assume that σ_S_ remains constant with change in ionic strength. These values and assumptions allow us to extract surface charge and contact distance from Eq. 4. While the van der Waals force is not explicitly calculated in order to fit the data, the measured adhesion forces are consistent with Hamaker constants for the interaction between an alkane functionalised tip and a alkane functionalised substrate (Hamaker constant: ≈ 6.2 × 10^−21^ J) or a quartz surface (Hamaker constant: ≈ 1.2 × 10^−20^ J) which would give F_vdW_ that lies between 30 pN and 60 pN for a contact distance of 1 nm.

From the pooled data of Figure 4D, we determined the surface charge to be −0.015 ± 0.012 C/m^2^ and the contact distance to be 1.0 ± 0.6 nm. Uncertainties were derived from the standard deviation for the fit. The surface charge of pure, clean quartz depends weakly on ionic strength. For comparison, Zheng et al.^38^ reported −0.00885 C/m^2^ at pH 6 and ionic strength of 0.001 M NaCl. Ebeling et al.^39^ reported −0.01 C/m^2^ at pH ~ 5 in 0.0094 M NaCl for a silicon wafer surface, which is certainly amorphous SiO_2_. Milonji'c^40^ reported −0.0094 C/m^2^ in 0.01 M NaCl and −0.02 C/m^2^ in 0.5 M NaCl at pH 6.5 for SiO_2_ colloids. The surface of the sand grains is not pure quartz. Clay nanoparticles and organic material are also present. Therefore we estimated the surface charge of crude oil as well. At pH 5.5, the surface charge of oil depends on the chemical composition but we estimated it from carboxylic acid concentration and pK_a_ at oil/water interfaces^30^ to be about −0.007 C/m^2^ for a total acid number of 0.3 (details for the estimation procedure are in Supporting Information). The negative surface charge is consistent with the literature^41^. Divalent ions present in the brine can bind to the acids and decrease the negative charge or even reverse the charge. Either way, when high salinity solution is replaced with low salinity, the concentration of divalent ions decreases and in parallel, binding to crude oil acids decreases. Thus, the surface charge stays the same or increases in magnitude when salinity decreases.

From the experimental data, which range from −0.02 C/m^2^ for 0.5 M NaCl to −0.01 C/m^2^ for 0.01 M NaCl, we can extrapolate that surface charge on clean quartz changes by 0.01 C/m^2^ when ionic strength changes by a factor of 50. In our studies, the only significant change in the adhesion force occurs between ~ 8,000 and 1,000 ppm, namely a change in ionic strength by a factor of < 10. If we assume surface charge on quartz changes linearly with ionic strength, we can estimate a change of 0.002 C/m^2^, significantly less than the uncertainty in the fit. This offers support for our constant charge assumption. Equation 5 is only valid for D ≥ λ_D_ so our application is a borderline case; D = 1.0 nm, which is to say that 0.4 < λ_D_ <1.5 nm. Within the standard deviation of the charge from the fit, the charge could equally well come from organic material adsorbed on the quartz grain surface. This means that regardless of variation in the surface composition and adhesion across the surface, the tip interaction with both mineral and adsorbed organic material is affected by double layer repulsion.

**Figure 5:**
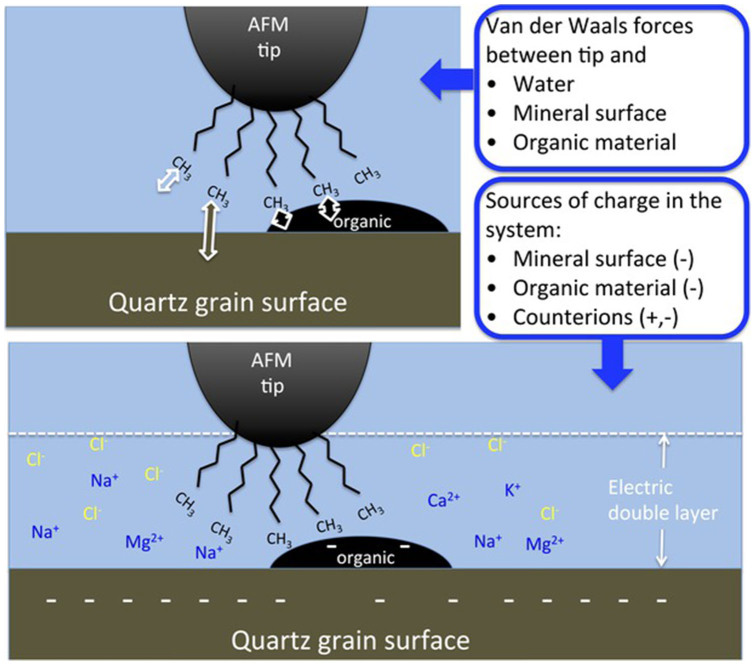
Schematic diagram of the interaction forces between the AFM tip and the quartz grain surface. The top panel shows the van der Waals forces between the tip and the various components in the system: water, quartz grain surface and adsorbed organic matter. The bottom panel shows the various sources of charge in the system, which contribute to the EDL repulsion. Important to note is that both the quartz grain surface and the organic material are negatively charged at low salinity and pH 5.5.

Even though the spread in experimental data is considerable, the fit to the relationship provides very reasonable values for surface charge and contact distance. This suggests that the electric double layer forces that we have considered in the simple model play a definitive role in the tip-surface interaction and could explain some of the decrease in adhesion of hydrocarbons to sandstone particle surfaces in low salinity solutions. We do not dispute that other mechanisms could be at play in the low salinity water flooding effect. What we have aimed to demonstrate here, is that if all other variables are kept constant, the adhesion between nonpolar oil compounds, such as alkanes and the real pore surface is directly correlated with ionic strength and the EDL thickness. A schematic figure of the bonding of the tip to the surface is shown in Figure 5. Given the heterogeneous nature of the surface on the nanometer scale, it is likely that parts of the tip are in contact with organic material, while still keeping an average distance to the surface of 1 nm, which is consistent with the value we derived from our fit to the data presented in Figure 4D.

Our studies were made with a tip functionalised to represent an oil droplet of alkane, i.e. nonpolar. In the literature, focus is put on adsorption of polar compounds to mineral surfaces, which is often facilitated by the presence of specific ions^5^,^9^. When the pores are flooded with a low salinity solution, surface and solution must re-equilibrate, which changes surface composition, thus changing pore surface wettability. There is evidence that reduced salinity water flooding through a sample containing oil without polar compounds does not result in increased oil production for certain wetting conditions^42^, unless the migration of fines play a key role^43^. However, our results show that adhesion of nonpolar compounds is also affected by salinity changes, implying that at least part of the mechanism for decreased adhesion with decreased salinity is independent of the presence of specific ions. All that is needed for the adsorption/desorption of an alkane oil droplet, such as described in this paper, is a charged pore surface, with a surface composition that makes the nonpolar oil droplet adhere. Nonpolar oil does not adhere well to clean mineral surfaces such as quartz but it adheres well to a mineral that has been aged in crude oil with polar components^33^. Our results also demonstrate that adhesion remains constant at high ionic strength but as salinity decreases, a threshold is reached, in the range of ~ 5000 ppm, where it decreases. This threshold range matches remarkably well with observations in core plug and field tests^1^,^2^,^44^,^45^. It corresponds to a Debye length of about 1 nm.

The presence of organic material with polar components including acids does not change the surface charge by much. However, without polar components, the crude oil is not able to change the wettability of quartz^33^. Thus, polar components must be part of the ageing oil, to develop a surface to which oil can adhere and therefore, polar compounds are necessary for conditioning a mineral surface. If the organic material that is deposited on the pore surface has a high enough concentration of acids, the surface charge of the particle could be higher than that of quartz because of the adsorbed organic material. This would explain why the adhesion change as the solution is changed from high to low salinity was larger on areas with higher adhesion, i.e. more organic material^23^.

The clay minerals present in sandstone generally have higher surface charge than quartz. The surface charge of kaolinite at pH 5.5 is at least an order of magnitude higher than for quartz at the same pH^46^. This implies that if the constant charge model is also valid for clays present in sandstone, the repulsion of alkanes caused by expansion of the electric double layer at clay surfaces could be much higher, as much as 500 pN at 5000 ppm salinity. While there is some evidence from core flood tests^42^ that clays are necessary for a low salinity effect, our results suggest that even the interaction of nonpolar organic compounds with sand grain surfaces is affected by decreased salinity. Clays also have a high affinity for organic material. Thus it is also possible that acidic or basic organic compounds from the oil could be strongly attached at the surface, thus also changing the surface charge and adhesion properties. There is evidence that clay nanoparticles^20^ and organic material^23^ are present on quartz, which certainly play a role in adhesion. Both the higher charge and higher affinity for organic material suggest that the low salinity effect should be more easily observed when clays are present. Our observation, that electric double layer expansion is an important part of the low salinity mechanism, is quite consistent with both the fact that polar compounds (wettability alteration) and clays (wettability and higher charge) are necessary to observe a low salinity effect.

EDL expansion is a consistent explanation for decrease in adhesion measured using AFM and is an effect that would always be present during flooding in pores where charged surfaces provide sites for oil to stick. At pH 5.5, the quartz mineral surfaces is negatively charged and in a reservoir rock, crude oil has been present for a long time and has modified the pore surface composition from initially water wet to mixed wet. Thus, EDL expansion is expected in low salinity flooding in all sandstones. It is possible that the magnitude of adhesion decrease that we observe on the quartz grain surfaces is too low to have an effect at the macroscopic scale and that clays are needed either to increase pore surface charge through their composition or to provide a higher surface area and an anchor point for the oil compounds, thus increasing the effect of the EDL expansion.

### Summary and conclusions

Using an AFM tip functionalised with alkanes to model a nonpolar oil droplet, we probed the surfaces of individual quartz grains from a sandstone that is known to respond moderately well to low salinity flooding. Using force mode AFM, we collected adhesion force maps in a series of solutions where salinity was sequentially diluted from artificial formation water to low salinity artificial sea water to mimic a low salinity flood, as is used in EOR.

Adhesion force began to decrease at a threshold salinity that ranged from 5000 to 8000 ppm, which matches remarkably well with the results of core plug experiments and field tests.

We fit the results with theory for electric double layer forces between the tip and sample. Below the threshold, the repulsive force from the electric double layer was strong enough to significantly decrease total attraction between the tip and the sample so theory predicted lower adhesion, a decrease in oil wettability, as we observed. The fit provides very reasonable estimates of surface charge and tip-surface contact distance. For polar molecules, adsorption processes are likely to be much more complex but the results presented here are generally applicable. We demonstrate that the electric double layer force is always at least a part of the low salinity effect. By designing the experiments with an alkane functional group, we were able to prove that the electrical double layer has an effect on the release of oil from quartz surfaces, even when the oil molecule is uncharged. We predict that the ionic strength effect is at least one order of magnitude greater on clay surfaces than on quartz, as a result of the much higher surface charge on the type of clay minerals usually observed in sandstone.

## Author Contributions

S.L.S.S. and T.H. had the idea and planned the experiments. E.H. performed all experiments and prepared Figures 1–3, M.P.A. performed the theoretical analysis and prepared Figures 4 and 5. E.H. and M.P.A. wrote the manuscript with contributions by T.H., J.M., P.A.S. and S.L.S.S. All authors contributed to scientific discussions.

## Supplementary Material

Supplementary Information Supplementary information

Supplementary Information PDF Manuscript file with markup shown
