# Supplementary material for: The effect of ionic strength on oil adhesion in sandstone – the search for the low salinity mechanism
Source: Sci Rep. 2015 Apr 22;5:9933. doi: 10.1038/srep09933 (PMC4405700; doi:10.1038/srep09933)
Supplement: Supplementary Information [file srep09933-s1.doc]

**Supporting information for:**

**The effect of ionic strength on oil adhesion in sandstone – the search for the low salinity mechanism**

E. Hilner^1^, M. P. Andersson^1,*^, T. Hassenkam^1^, J. Matthiesen^1^, P. A. Salino^2^ and S. L. S. Stipp^1^

^1^ *Nano-Science Center, Department of Chemistry, University of Copenhagen, Denmark.*

^2^ *BP Exploration, Sunbury, UK.*

** corresponding author:* [ma@nano.ku.dk](mailto:ma@nano.ku.dk)

**Additional figures:**


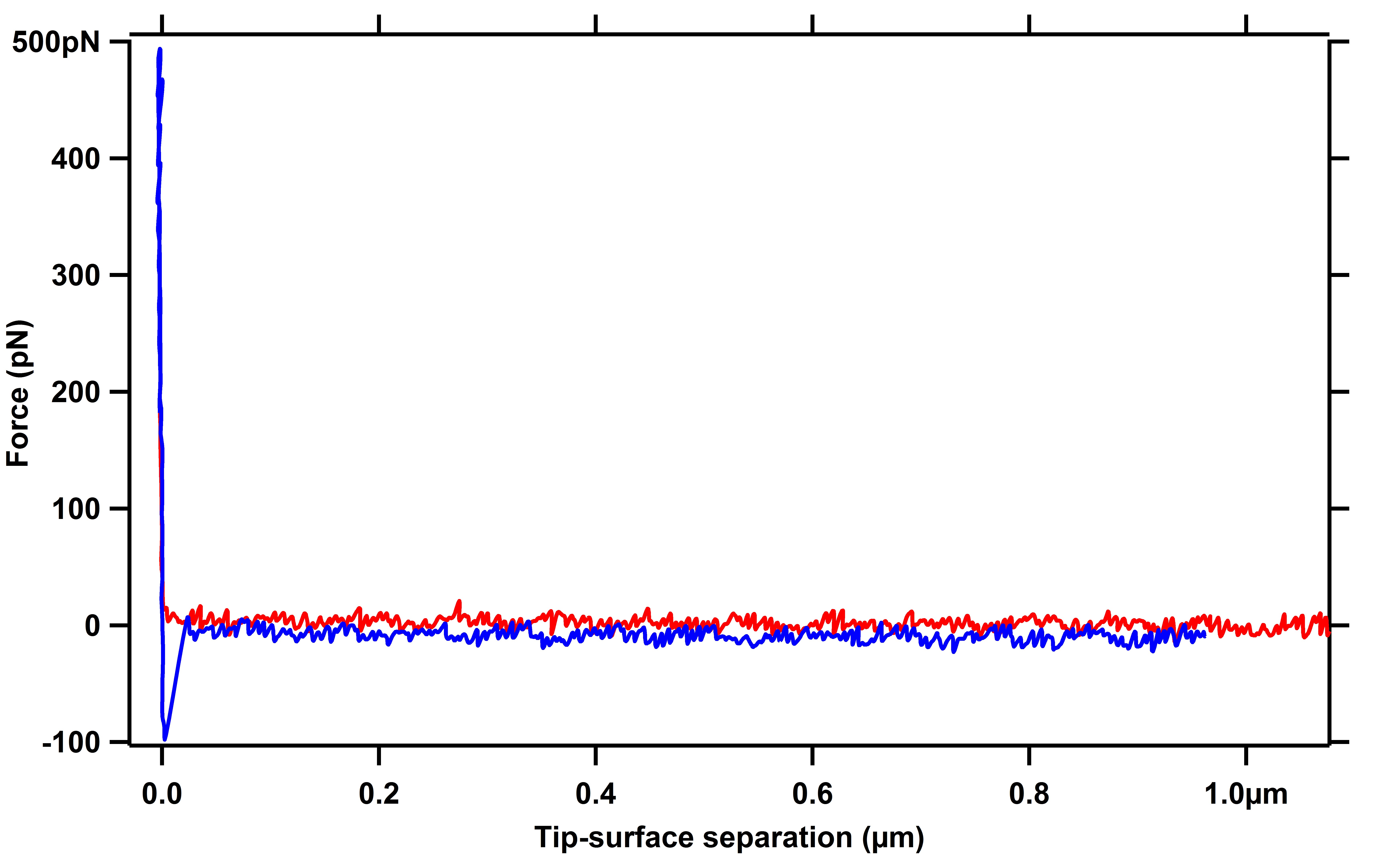


Figure S1: Force-distance curve measured with a functionalized AFM tip. The red curve marks the tip’s approach and the blue curve, the withdrawal from the surface. The small offset (10-20 pN) between the approach and withdrawal curves results from viscous drag as the cantilever moves through the liquid. The tip is pushed toward the surface until the maximum force, 0.5 nN, is reached. The adhesion force is measured as the tip springs free of the surface during retraction, in this case, about 100 pN.

**
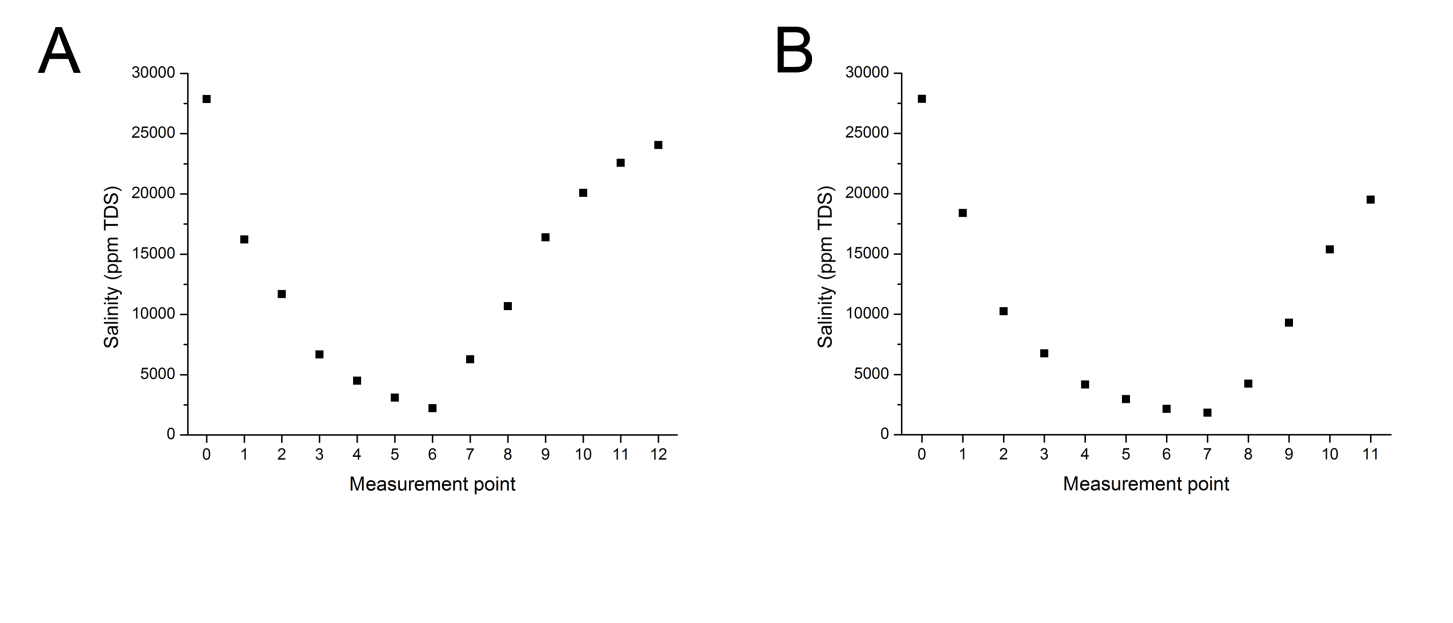
**

**Figure S2:** Salinity derived for the measurement points in A) Experiment 2 and B) Experiment 3. In both cases, solutions were synthesised to match the composition of original formation water (AFW, artificial formation water, Point 0) and then diluted with low salinity, ASW (artificial sea water), to Point 6. Then concentration was increased again using aliquots of AFW.


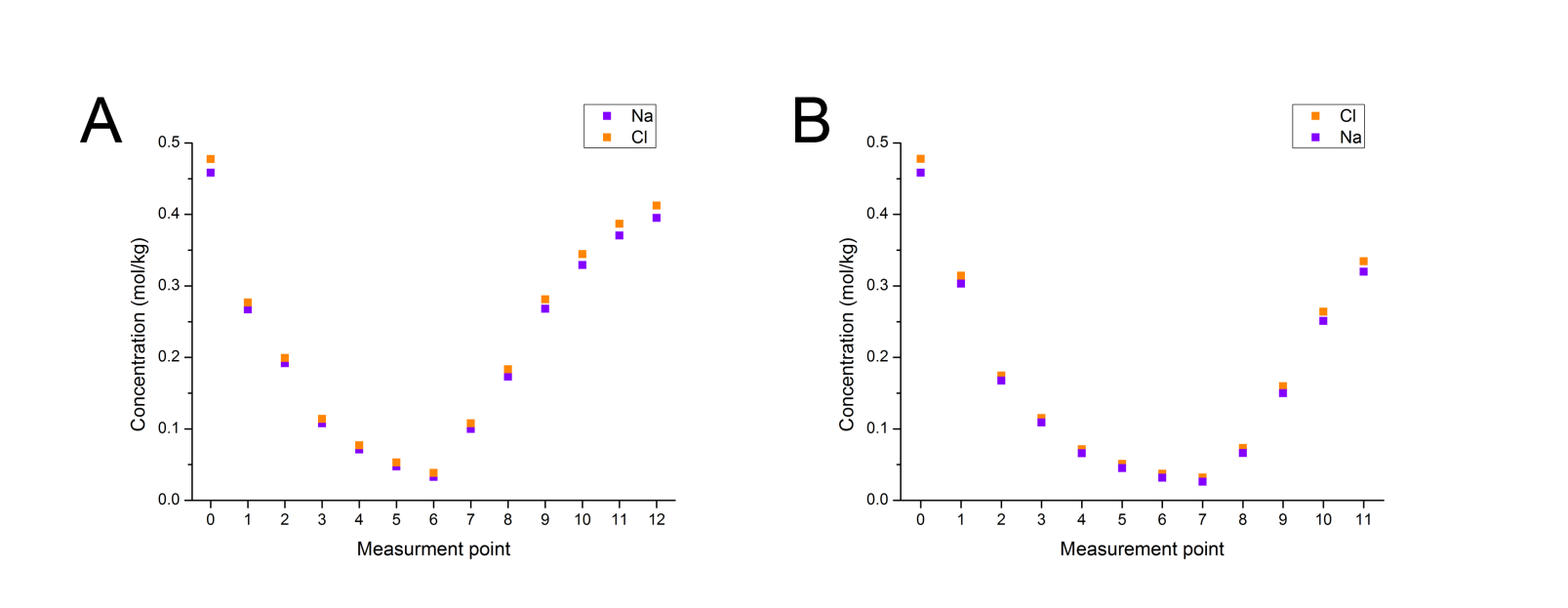


**Figure S3:** Concentrations of Na and Cl from Atomic Absorption Spectroscopy (AAS) analysis for the solutions of A) Experiment 2 and B) Experiment 3. Point 0 in both cases corresponds to the initial solution, synthesised to represent pure formation water.

**
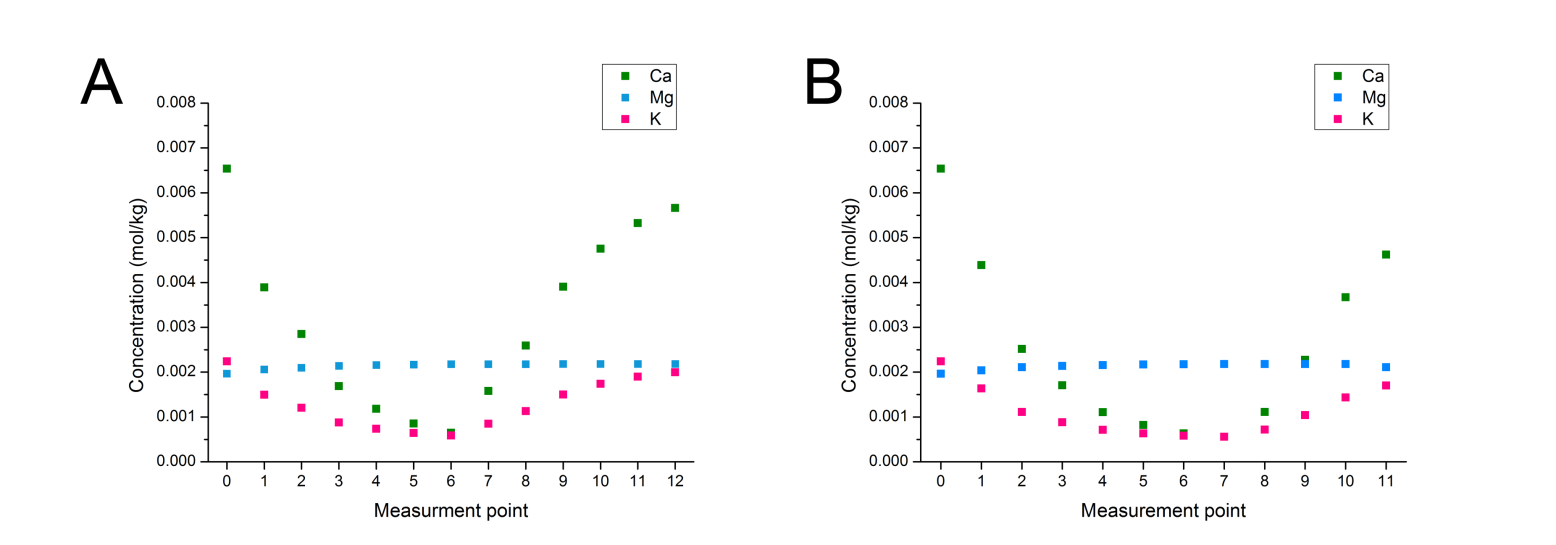
**

**Figure S4:** Concentrations from AAS analysis for Ca, Mg and K in A) Experiment 2 and B) Experiment 3.

**Estimation of surface charge of crude oil**

We have recently predicted that pKa values of organic acids at an oil/water interface are shifted compared to their bulk values [^1^](#_ENREF_1). For aliphatic as well as aromatic carboxylic acids, the interfacial pKa is about 6. Of all common functional groups present in crude oil, carboxylic acids are the ones that most enrich at the oil/water interface and define the surface charge. For a total acid number (TAN) of 0.3, the surface mole fraction of carboxylic acids is about 12%,[^1^](#_ENREF_1) assuming that the average molecular weight is ~200 g/mol. The ratio between deprotonated acids and protonated acids at the interface is:

$$\frac{\left[A^{-}\right]}{\left[HA\right]}=\frac{10^{-pK_{a}}}{10^{-pH}}$$

which amounts to 0.32 for pH = 5.5. This gives a surface coverage of charged molecules of 3.8%. We assume that the oil density is 900 g/dm^3^ , that all molecules are spheres and that the area taken up at the oil/water interface by molecules is the maximum cross sectional area of a sphere. This gives a surface area per molecule of 6.2e-19 m^2^. Together with the 3.8% coverage of singly, negatively charged acid groups, we obtain a surface charge of about -0.007 C/m^2^.

1. Andersson, M. P.; Olsson, M. H. M.; Stipp, S. L. S., Predicting pKa and stability of organic acids and bases at an oil-water interface *Langmuir* **2014,** 30, (22), 6437-6445.
